# A novel QTL associated with dwarf bunt resistance in Idaho 444 winter wheat

**DOI:** 10.1007/s00122-016-2783-2

**Published:** 2016-09-28

**Authors:** Jianli Chen, Mary J. Guttieri, Junli Zhang, David Hole, Edward Souza, Blair Goates

**Affiliations:** 1 University of Idaho, 1693 S 2700 W, Aberdeen, ID 83210 USA; 2 USDA, Agricultural Research Service, Center for Grain and Animal Health Research, Hard Winter Wheat Genetics Research Unit, 4011 Throckmorton Hall, Manhattan, KS 66506 USA; 3 Department of Plant Sciences, University of California, Davis, One Shields Avenue, Davis, CA 95616 USA; 4 Utah State University, 2325 Old Main Hill, Logan, UT 84322 USA; 5 Bayer Crop Science, Beaver Crossing, NE 68313 USA; 6 USDA-ARS (retired), Aberdeen, ID 83210 USA

## Abstract

***Key message*:**

**A novel QTL, Q.DB.ui-7DS, and the PCR-based markers identified in the current study will accelerate variety development for resistance to dwarf and common bunt of wheat.**

**Abstract:**

Dwarf bunt [*Tilletia controversa J.G. Kühn [as ‘contraversa’], in Rabenhorst, Hedwigia 13: 188 (1874)*] is a destructive disease of wheat (*Triticum aestivum* L.) that reduces grain yield and quality. A number of distinct genes conferring resistance to dwarf bunt have been used by breeding programs for nearly 100 years. However, few markers were identified that can be used in selection of dwarf bunt resistance. A recombinant inbred line (RIL) population derived from the bunt-resistant germplasm, Idaho 444 (IDO444), and the susceptible cultivar, Rio Blanco, was evaluated for phenotypic reaction to dwarf bunt inoculation in four trials in two locations (USU and USDA) over 3 years. The population was genotyped with the Diversity Arrays Technology (DArT) and the Illumina Infinium 9K iSelect marker platforms. A total of three QTL were detected, and resistant alleles were from IDO444. QTL *Q.DB.ui*-*7DS* on 7DS was determined based on the location of a DArT marker *wPt*-*2565* (*X116197*), which was consistently detected and explained 32 to 56 % of phenotypic variation among the four trials. QTL *Q.DB.ui*-*1A* on 1A was detected in three Utah State University (USU) trials and explained 11–15 % of phenotypic variation. QTL *Q.DB.ui*-*2B* on 2B was detected in two USU and one United States Department of Agriculture (USDA) trials and explained up to 6 % of phenotypic variation. Two PCR-based markers were developed based on the sequence of *wPt*-*2565* and validated in the RIL population and used in genotyping of dwarf bunt differential lines, known resistance sources, and resistant cultivars.

## Introduction

Although Mendel did not directly investigate the relationships of disease and resistance in plants, a significant component of his legacy has been the ability to discover and utilize genes for resistance to diseases in crops. Dwarf bunt caused by [*Tilletia controversa J.G. Kühn [as ‘contraversa’], in Rabenhorst, Hedwigia 13: 188 (1874)*], and common bunt caused by *Tilletia caries* (DC.) Tul. & C. Tul. (= *T. tritici*) and *T. foetida* (Wallr.) Liro (= *T. laevis*) are two destructive diseases of wheat (*Triticum aestivum* L.) that reduce grain yield and quality because of the formation of sori, called bunt balls, that replace the grain with brown–black spores with an unpleasant odor (Cherewick 1953; Martens et al. 1984). While no mycotoxins have been identified from common bunt or dwarf bunt sori, these sori, composed almost entirely of teliospores, contain significant levels of trimethylamine, which has a strong odor of rotting fish, and can contaminate flour. Even relatively low infection rates can result in noticeable odors in flour milled from infected wheat.

The three *Tilletia* species that cause these two wheat bunt diseases are closely related, evidenced by the inability of modern molecular techniques to differentiate them when sufficient numbers of isolates are compared (Bao, et al. 2011; Bao and Carris 2009). The fungi are related to the extent that both dwarf and common bunt host plant resistances (HPR) are controlled in wheat by the same genes (*Bt*) in a classic gene-for-gene system of host–pathogen interaction (Metzger and Hoffmann 1978; Goates 1996, 2012). Thus, HPR genes effective against dwarf bunt also confer resistance to common bunt. Currently, 36 pathogenic races of *T. caries*, 15 races of *T. foetida* and 19 races of *T. controversa* are identified based on their reaction to 14 wheat differential lines that each putatively contains one of 14 recognized bunt resistance genes, *Bt1* through *Bt13*, and *Btp* (Goates 2012). Two additional HPR genes, *Bt14* and *Bt15*, were identified by R.J. Metzger in the tetraploid (durum) spring habit wheats Doubbi (CI 13711) and Carleton (CI 12064), respectively (Goates 1996). They were excluded from recent pathogenic race tests primarily because they demonstrated temperature-sensitive responses in these wheat backgrounds. Thus, they may not reliably give a correct resistant or susceptible reaction to defined bunt races (Goates 2012).

Several fungicide seed treatments are effective against common bunt, and one seed treatment, difenoconazole, is currently labeled for control of dwarf and common bunt in the US. Yet, host resistance is an important part of cultivar development in many areas of the world. In the US, low-input rain-fed farms in the Intermountain West rely on HPR to control dwarf bunt, and a high level of resistance is maintained in cultivars from these breeding programs. In Western Canada, common bunt is listed as a Priority 1 disease in the registration testing system. As a result, bread wheat varieties registered in Canada are expected to have a minimum intermediate resistance reaction to common bunt (http://pgdc.ca/pdfs/wrt/2012–2013%20PRCWRT%20Operating%20Procedures.pdf). With the introduction of effective seed treatments, most breeding programs de-emphasized common bunt and dwarf bunt HPR selection. However, bunt HPR has a renewed world-wide interest due to the increase in organic farming and concern for sustainable agriculture (Matanguihan et al. 2011). With few current options for organic certified seed treatments for control of common bunt, organic wheat production has experienced increasing incidence of this disease. Development of organic certified seed treatments for dwarf bunt is more difficult than for common bunt due to the timing of infection and the necessity of systemic anti-fungal activity that can persist throughout a lengthy infection period.

The dwarf bunt-resistant germplasm PI 178383, identified in the 1950s, has been widely used in variety development for common and dwarf bunt resistance. The PI 178383-derived cultivars released in the 1970s practically eliminated the disease in areas, such as the U.S. Intermountain West, where the disease was historically the most severe. The resistance genes from PI 178383, *Bt8*, *Bt9*, *Bt10*, and possibly an unidentified factor (Goates, unpublished), remain effective for controlling common bunt and dwarf bunt in the US. *Bt8* is likely responsible for the high level of resistance to dwarf bunt in some cultivars deriving resistance from PI 178383. It is the only known gene from that germplasm that is not compromised by known races of dwarf bunt in the U.S. (Goates 2012). The longevity of the resistance provided by PI 178383 demonstrates the durability of the resistance to bunt that can be provided by specific genes. A different source of resistance that comes from the gene *Bt12*, which originated from CI 14106, has been utilized in newer cultivars and has been durable since the release of the cultivars beginning in the early 1990s (Souza, et al. 1992; Hole et al. 2002). PI 476212, a snow mold-resistant line, also was a source of dwarf bunt resistance (Sunderman et al. 1986, 1991). Although the bunt resistance in these wheat cultivars has been durable in the US, they represent a narrow genetic basis of three resistance sources for disease control. Recently, highly effective sources of resistance that contain genes or gene combinations that are different from all other known resistant sources were identified in landraces in the USDA-ARS National Small Grains Collection (Goates and Bockelman 2012). The resistance gene(s) in these lines have yet to be characterized.

Previously, identification of genes present in breeding lines and cultivars relied on their reaction to specific pathogen races that have been characterized by their virulence on the current set of differential lines. There is some evidence that the current differential set of bunt lines may, in fact, not always be monogenic. Race D-18 expresses weak virulence to the *Bt8* differential line, and L-18 and D-19 express virulence to the *Bt8* differential line. Race D-7 lacks virulence to the *Bt8* differential, but it is virulent on CI 9342 and PI 636146, which putatively contain *Bt8* due to their reaction to L-18, D-18, and D-19 (Goates 2012). One explanation for this is that there may be an additional minor HPR gene in the *Bt8* differential.

Assessment of common and dwarf bunt HPR in the field is performed at plant maturity. Dwarf bunt can be difficult to reliably induce due to stringent environmental requirements that include several weeks of stable cool soil temperatures, a moist environment at the soil surface, and low light levels. These conditions are most reliably provided by continuous snow cover and are critical for teliospore germination. Molecular markers for specific HPR genes would minimize effects of the variability in phenotyping due to the seasonal vicissitudes and would help in both characterizing and pyramiding resistance genes in breeding lines. Molecular markers are also useful to further characterize the differential lines for identifying bunt races. However, QTL mapping of bunt HPR is far behind other traits in wheat. One QTL on 1BS for common bunt was reported in two spring wheats, AC Domain (Fofana et al. 2008) and Carberry (Singh et al. 2016), a US winter wheat Blizzard (Wang et al. 2009), and a European winter wheat Trintella (Dumalasová et al. 2012). Additional QTL were reported on 7A (Fofana et al. 2008; Dumalasová et al. 2012), 7B (Dumalasová et al. 2012; Knox et al. 2013), 5B (Dumalasová et al. 2012; Singh et al. 2016), 4D (Singh et al. 2016), 6D (Menzies, et al. 2006; Singh et al. 2016), and 7D (Singh et al. 2016). Markers for *Bt10* on 6D have also been developed (Laroche et al. 2000; Menzies et al. 2006).

The objective of this study was to identify QTL and molecular markers associated with the dwarf bunt resistance in ‘Idaho 444′ (IDO444, PI 578278, Windes et al. 1995). IDO444 demonstrated high levels of resistance to dwarf bunt during its years of testing, but was not released as a cultivar due to its inferior milling and baking quality.

## Materials and methods

### Plant materials

The mapping population used in this study consisted of 159 F_8:10_ recombinant inbred lines (RILs) derived from a cross of ‘Rio Blanco’ to IDO444 (Rio Blanco/IDO444). Rio Blanco (PI 531244) is an early maturing, semi-dwarf (Rht-B1b, Rht-D1a) hard white winter wheat cultivar released by Agripro Biosciences, Inc., Shawnee Mission, KS (Wu and Carver 1999). IDO444 (PI 578278) is a tall (Rht-B1a, Rht-D1a) hard red winter wheat germplasm developed by University of Idaho in Aberdeen, ID. IDO444 has HPR to dwarf bunt (caused by *Tilletia controversa J. G. Kühn*), snow mold (caused by *Typhula spp*.) (Windes et al. 1995), and high temperature adult resistance to stripe rust (caused by *Puccinia striiformis f. sp. tritici)* (Windes et al. 1995; Chen et al. 2012). However, it has poor pan bread quality (Windes et al. 1995). IDO444 had longer coleoptiles (Li et al. 2011) and greater grain yield than Rio Blanco in rain-fed conditions (Zhang et al. 2014).

### Disease evaluations

Phenotypic disease evaluations were conducted at two locations over 3 years: the Utah State University (USU) Research Farm in Logan, UT (41°45′46.46″N, 111°48′54.98″W, elevation: 1400 m) in 2003, 2004 and 2011 (hereafter USU03, USU04, USU11), and the USDA-ARS disease screening nursery in Green Canyon (approximately 3 km east of Logan; 41°46′21.05″N, 111°46′52.68″W. elevation: 1450 m) in 2003 (hereafter, USDA03). Both locations are known for having long periods of snow cover, which is essential to induce high levels of disease. The nurseries were inoculated after seedling emergence, prior to snow cover with water suspension of dwarf bunt spores. Sowing for all locations was timed early in the autumn (usually the end of September). Approximately 100-ml water spore suspension per m row was used to inoculate the individual rows in early to mid-November. Generally, this resulted in between 200 and 300 million spores applied per m row. The USU nursery inoculum originated from locally collected diseased spikes from commercial fields. The USDA inoculum consisted of a broad composite of field collections and known pathogenic races that originated from throughout the Pacific Northwestern United States. To maintain inoculum for both locations, diseased spikes were harvested from susceptible and partially resistant lines, in addition to diseased spikes from separate race increases and other studies that utilized specific races. In all cases, the inoculum for a particular location-year originated with spores collected from the previous year. Parental lines and RILs were planted in rows that were 1 to 2 m in length, replicated twice in a randomized complete block design in all trials. Disease incidence was estimated as a percentage of bunted spikes per row at plant maturity (Zadoks stage 92, Zadoks et al. 1974).

### Phenotypic analysis

Spearman’s rank correlation analysis was conducted using R (R Core Team 2015). Broad sense heritability $(h_{B}^{2} )$ was calculated by fitting a mixed linear model using R package "lme4" (Bates et al. 2015; R Core Team 2015). Genotype and replication were fitted as random effects to estimate $h_{B}^{2}$ according to equation $h_{B}^{2} = \sigma_{G}^{2} /(\sigma_G^{2} + \sigma_e^{2}/r )$ for each single environment, where $\sigma_{G}^{2}$ is genetic variance, $\sigma_{e}^{2}$ is error variance and *r* represents the number of replications.

Best linear unbiased predictors (BLUP) of dwarf bunt incidence across four environments were obtained from a mixed linear model using R package "lme4" (Bates et al. 2015; R Core Team 2015). Genotype, environment and replication were all treated as random effects in the model. Broad sense heritability was estimated according to equation $h_{B}^{2} = \sigma_{G}^{2} /(\sigma_{G}^{2} + \sigma_{GE}^{2} /r + \sigma_{e}^{2} /re)$, where $\sigma_{G}^{2}$ is the variance of genotypes, $\sigma_{GE}^{2}$ is the variance of genotype by environment interaction, $\sigma_{e}^{2}$ is the residual variance, *e* is environment number, and *r* is the number of replications in each environment. Likelihood ratio test of the variance components was conducted using the “rand” function in R package “lmerTest” (Kuznetsova et al. 2016).

### QTL analysis

The whole genome linkage map developed based on this RIL population was previously obtained, and the map included 739 markers with the average density of 6.7 cM per marker, representing all the 21 chromosomes except for 1D, 5D, and 7D (Chen et al. 2012). The population was later genotyped by 9K single nucleotide polymorphism (SNP) markers from the IIlumina Infinium 9K iSelect platform (Cavanagh et al. 2013) in the USDA-ARS genotyping lab at Fargo, North Dakota. By adding 413 SNPs to the previous map, the average interval between two markers was reduced from 6.7 to 3.4 cM, which excluded markers with high segregation distortion (χ^2^ test at α = 0.01). The maps were constructed using software MSTmap (http://alumni.cs.ucr.edu/~yonghui/mstmap.html) (Wu et al. 2008) and Mapmaker/EXP 3.0b (Lander and Botstein 1989). The SNP names in the map were “IWA” (Illumina wheat Design A) plus the index number of the SNP, such as “IWA7179″. The full SNP names and indexes can be accessed from Cavanagh et al. (Cavanagh et al. 2013). The marker groups and the marker order in each group were determined in MSTmap. The marker orders were checked in Mapmaker 3.0b using the “ripple” function. Map distances were calculated using Kosambi function in Mapmaker and given in centimorgan (cM). Updated maps were used in QTL detection of grain yield (Zhang et al. 2014) and HPR to dwarf bunt in the present study, and included 413 SNPs, 342 DArTs, 106 SSRs, and 1 sequence-tagged-site (STS) marker from the semi-dwarf gene *Rht*-*B1*, representing all the 21 chromosomes except 1D and 5D.

The mean bunt incidences of each RIL, in each trial, were used separately in QTL analysis using R/qtl package (Broman et al. 2003). The function “scanone” with option method = “hk” identified single QTL genome widely with a threshold LOD = 2. A multiple QTL model was fit with the QTL identified in the single QTL scan using the function “fitqtl”. Only significant QTL and QTL x QTL interactions (LOD >2) were reported. Variation explained (*R* ^*2*^) by each QTL and the whole model were obtained from the multiple QTL model. Genomic regions of the corresponding QTL were determined with the 1-LOD support interval method. Genetic maps and QTL were drawn using Mapchart v2.2 (Voorrips 2002).

### Marker development and validation

The BLASTN function was used to determine the assignment of DArT markers based on the alignments identity in the Ensemble wheat genome assembly (IWGCS1 + popseq) in the EnsemblePlants browser (http://plants.ensembl.org) and the TGACv1 wheat genome assembly in the URGI genome browser (http://wheat-urgi.versailles.inra.fr/Seq-Repository/BLAST). The online computer program Primer 3 (http://frodo.wi.mit.edu/primer3/) was used to design primers to amplify the sequence of the identified DArT markers associated with the traits of interest. The designed sequence targeted site (STS) primers were screened using the parental lines and genotyped in the RILs. STS primers also were used in marker-assisted evaluation in fifteen dwarf bunt differential lines, four known sources of resistance, 11 resistant cultivars, and one susceptible check. These additional lines were also genotyped using the DArT platform.

## Results

### Phenotypic analysis of dwarf bunt incidence

**Fig. 1:**
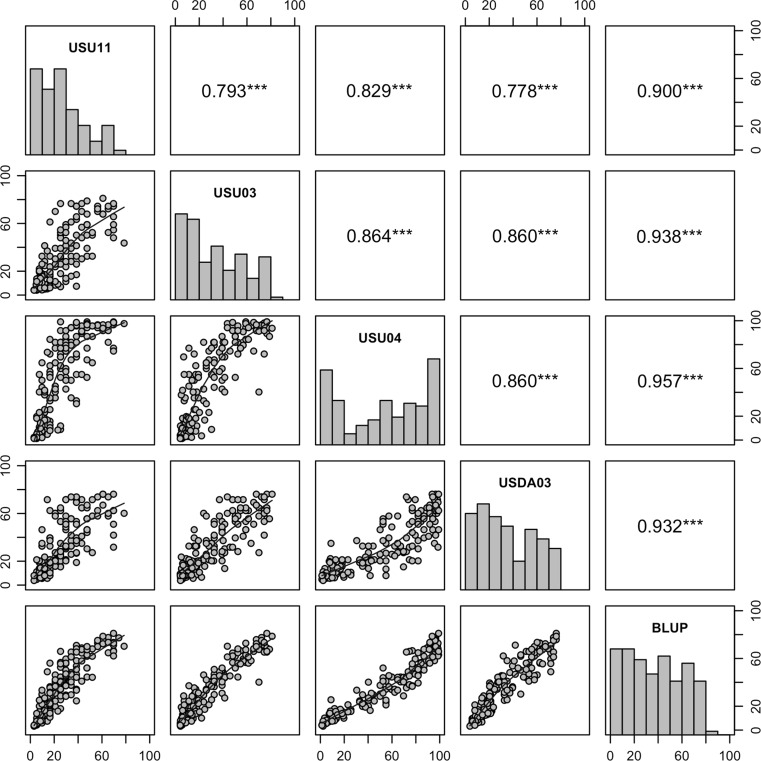
Distribution and correlation of dwarf bunt incidence (%) in the Rio Blanco x IDO444 population among individual environments and the best linear unbiased predictors (BLUP) over the four environments. The diagonal contains histograms of dwarf bunt incidence in each environment, scatterplots with a Lowess smoothing line between each environment in the lower diagonal, and the Spearman’s rank correlation coefficient in the upper diagonal with significance test (*triple asterisk* indicates significance *p* < 0.001)

Parental lines and RILs had significant genetic variation for HPR reaction to the dwarf bunt inoculation. The resistant parent, IDO444, and the susceptible parent, Rio Blanco, always demonstrated low and high disease incidence, respectively (Table 1). The distribution of bunt incidence in RILs (Fig. 1) was negatively skewed in all environments except USU04. The genetic repeatability as estimated by broad sense heritability of dwarf bunt incidence was high (88–98 %) in all field trials (Table 1). Although environment variance was significant (*p* = 0.01), it was much smaller than genotypic variance (*p* < 2e-16) (Table 2). Correlation coefficients were high (0.78 to 0.96) between the four trials (Fig. 1). The genetic repeatability as estimated by broad sense heritability across the four environments was high (0.93, Table 1). Therefore, BLUP of bunt incidence were estimated from the four trials and were utilized in HPR QTL detection along with the mean bunt incidence of RILs in each trial.

**Table 1:** Dwarf bunt incidence of parents and 159 recombinant inbred lines (RILs) in four field trials and the best linear unbiased predictors (BLUP) over the four trials, and broad sense heritability $(h_{B}^{2} )$ of dwarf bunt incidence

Field trial	Parents		RILs				$(h_{B}^{2} )$
	Rio Blanco	IDO444	Min.	Median	Max.	Mean	
USDA03	87	1	1	31	80	35	0.92
USU03	90	1	0	28	88	33	0.88
USU04	100	1	1	55	100	53	0.98
USU11	80	5	0	25	85	28	0.89
BLUP	90	2	3	35	81	37	0.93

**Table 2:** Variance components of dwarf bunt incidence (%) in four field trials

Effect	Variance	*p* value of likelihood ratio test
Genotype	562.2	<2e-16
Environment	119.1	0.01
Genotype x environment	119.4	<2e-16
Environment x replicate	11.1	1.00e-12
Residual	104.0	

### QTL analysis

**Fig. 2:**
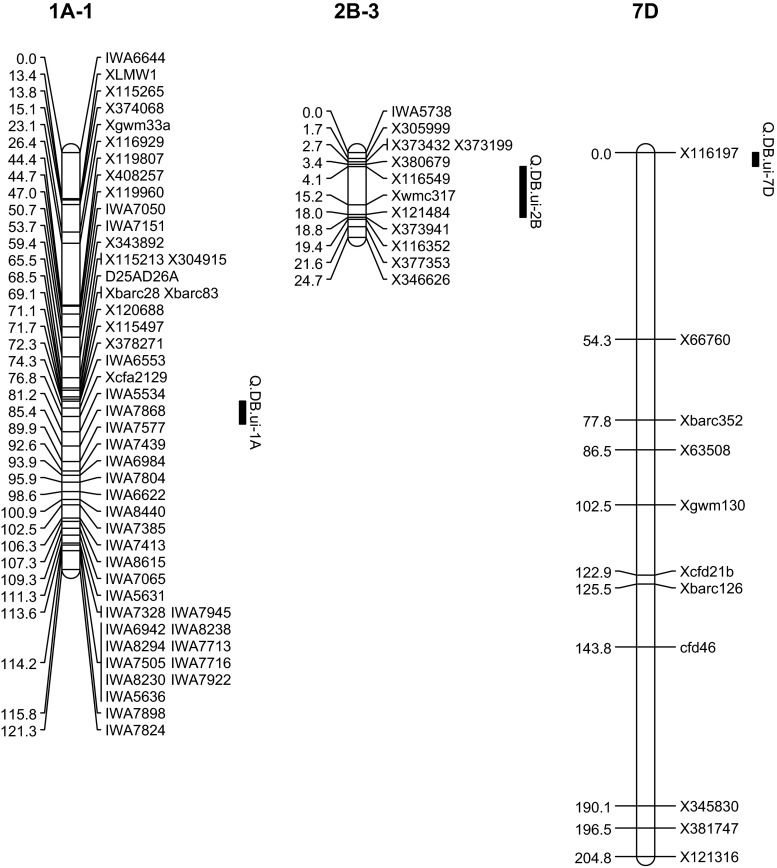
Linkage maps showing QTL associated with dwarf bunt resistance

A total of three HPR QTL were detected (Table 3 and Fig. 2). QTL *Q.DB.ui*-*7DS* on 7DS was determined based on the location (Wenzl et al. 2008) of a DArT marker *wPt*-*2565* (derived from DArT marker *X116197,* see http://www.diversityarrays.com/dart-map-sequences), which was consistently detected and explained 41 to 56 % of phenotypic variation among the four trials. QTL *Q.DB.ui*-*1A* on 1A was detected in three trials and explained 11–15 % of phenotypic HPR variation. QTL *Q.DB.ui*-*2B* on 2B was detected in three trials and explained up to 6 % of phenotypic variation. The three HPR QTL also were detected in the BLUP derived from all four trials.

**Table 3:** Main and interaction effect of dwarf bunt incidence QTL identified in Rio Blanco × IDO444 recombinant inbred population

Trial	QTL	Position (cM)	Peak marker	LOD	Effect	*R* ^2^ (%)	Total *R* ^2^ (%)
USDA03	2B	14	Xwmc317	3.9	5.9	5.5	56.0
	7D	1	wPt-2565	43.5	16.2	43.5	
USU03	1A	74	Xcfa2129	10.6	7.7	13.7	64.7
	2B	15.2	Xwmc317	3.8	5.8	4.4	
	7D	1	wPt-2565	28.1	17.7	48.8	
	1A × 7D	–	–	4.1	5.2	4.9	
USU04	1A	76	Xcfa2129	8.1	11.0	10.9	61.7
	7D	1	wPt-2565	29.0	25.7	55.6	
USU11	1A	76	Xcfa2129	10.7	7.2	15.0	58.6
	2B	13	Xwmc317	2.3	3.8	2.3	
	7D	1	wPt-2565	23.7	13.2	40.7	
	1A × 7D	–	–	3.0	3.8	3.8	
BLUP	1A	74.3	Xcfa2129	9.8	6.1	9.9	69.9
	2B	13	Xwmc317	3.7	5.0	3.7	
	7D	1	wPt-2565	35.2	16.5	53.4	
	1A × 7D	–	–	2.7	3.2	2.4

QTL × QTL interactions were detected, but were small (Table 3). The interaction effect between 7D × 1A only explained up to 5 % of phenotypic variation. The interaction effect of 7D × 2B and 1A × 2B were not significant. It was not expected that analyses of three-way interactions would be reliable due to relatively small population size.

### STS marker development for *wPt*-*2565*

The assignment of the DArT marker to 7DS is supported by BLASTN alignments of the nucleotide sequence to the wheat genome assemblies. The nucleotide sequence of *wPt*-*2565 (X116197)* provided by Diversity Array Technologies (Andrzej Kilian, personal communication) aligned with 100 % identity to IWGCS_CSS_7DS_contig_172158 at 7D: 3370320–3371070 in the Ensemble assembly. The nucleotide sequence for *wPt*-*2565* also aligned with 100 % identity to TGACv1_scaffold_62490_7DS.

Using the online computer program Primer 3 (http://frodo.wi.mit.edu/primer3/) we designed eleven pairs of STS primers based on the sequences of the wPt-2565 and TGACv1_scaffold_62490_7DS. Two STS markers were co-segregating with *wPt*-*2565* in RILs and the two parents. *XUIDB7D*-*11* amplifies a large product, which may be easier to detect with an agarose gel system. However, it is a dominant marker. *XUIDB7D*-*4* amplifies co-dominant small PCR products, but they may be more difficult to detect using agarose gels. The primer sequences, PCR conditions, and expected PCR products were summarized in Table 4.

**Table 4:** UIDB7D STS primer sequences, PCR conditions, and expected PCR products

STS primer	Forward	Reverse	Tm (^o^C)	Expected product (bp)
X_UIDB7D-4_	GTACTCCAGGCTGGCTCATC	CAGTGATTGTGGCACCAGAG	58 °C	154
X_UIDB7D-11_	TACCACCTACTGCCCTCTGG	CTTCCAACAGGAACAGAGCA	55 °C	1053

### Genotyping of the *Q.DB.ui*-*7DS* in the 15 dwarf bunt differential lines

The alleles for resistance in the two STS markers *XUIDB7D*-*4* and *XUIDB7D*-*11* were present in six resistant differential lines for *Bt5, Bt8, Bt9, Bt10, Bt12*, and *Bt13*, while absent in three resistant differential lines for *Bt11*, *Bt14*, and *Bt15*. The two STS markers were also present in ten of sixteen known resistant sources or cultivars evaluated (Table 5).

**Table 5:** Haplotypes of the 7DS markers in sixteen winter wheat cultivars and germplasm and fifteen dwarf bunt differential lines

Line	Genes^a^	Dwarf Bunt^b^	wPt-2565^c^	UIDB7D-4^c^	UIDB7D-11^c^
Cultivars					
Blizzard (PI 512302)	Unknown	R	1	1	1
Bonneville (PI 557015)	Unknown	R	1	1	1
Golden Spike (PI 614813)	Unknown	R	0	0	0
Gary (PI 620632)	Unknown	R	0	0	0
Promontory (PI 555458)	*Bt*-*3, Bt*-*9, Bt*-*10*	R	1	1	1
Manning (CItr 17846)	*Bt*-*3, Bt*-*9, Bt*-*10*	R	0	0	0
Deloris (PI 631447)	*Bt*-*3, Bt*-*9, Bt*-*10*	R	0	0	0
Lewjain (CItr 17909)	*Bt*-*8, Bt*-*9, Bt*-*10*	R	1	1	1
Winridge (CItr 17902)	*Bt*-*8, Bt*-*9, Bt*-*10*	R	1	1	1
Stava	*Bt*-*8, Bt*-*9, Bt*-*10*	R	1	1	1
Utah-100 (PI 594920)	*Bt*-*3, Bt*-*9, Bt*-*10*	R	0	0	0
Cheyenne (CItr 8885)	S control	S	0	0	0
Resistance sources					
PI 476212 (SM4)	Unknown	R	1	1	1
PI 178383 (M69-19)	*Bt8, Bt9, Bt10* +	R	1	1	1
CI 14106	*Bt12*	R	1	1	1
CI 14107	*Bt12*	R	1	1	1
Differentials					
M85-4 (PI 554101)	*Bt1*	S	0	0	0
M85-6 (PI 554097)	*Bt2*	S	0	0	0
M81-2008 (CI 6703)	*Bt3*	S	0	0	0
CI 1558	*Bt4*	S	0	0	0
M82-2052 (CI 11458)	*Bt5*	R	1	1	1
Rio (CI 10061)	*Bt6*	S	0	0	0
Sel. 50077 (PI 554100)	*Bt7*	S	0	0	0
PI 173438/Eg (M82-2161)	*Bt8*	R	1	1	1
Elgin/PI 178383 (M90-387)	*Bt9*	R	1	1	1
Elgin/PI 178383 (M82-2102)	*Bt10*	R	1	1	1
Elgin/PI 166910 (M82-2123)	*Bt11*	R	0	0	0
PI 119333	*Bt12*	R	1	1	1
PI 181463	*Bt13*	R	1	1	1
CItr 13711	*Bt14*	R	0	0	0
CItr 12064	*Bt15*	R	0	0	0

^a^Putative resistance genes based on race specific reaction data from Goates (2012) and/or personal communication

^b^Dwarf bunt resistance phenotype was based on many years’ evaluation in Intermountain West trials. R and S indicate resistant and susceptible, respectively

^c^Haplotype 1 represents IDO444 marker allele, 0 for all non-IDO444 marker alleles

## Discussion

The broad sense heritabilities of the dwarf bunt incidence were greater than 88 %, suggesting that dwarf bunt incidence is a highly inheritable trait in this population. Dwarf bunt HPR in IDO444 was likely contributed by PI 476212 based on the pedigree, which was documented as a snow mold-resistant line used as a source of dwarf bunt HPR (Sunderman et al. 1986, 1991). Three QTL, *Q.DB.ui*-*7DS, Q.DB.ui*-*1A, and Q.DB.ui*-*2B,* associated with dwarf bunt incidence were mapped on chromosome 7DS, 1A, and 2B, respectively. QTL x QTL interaction was detected but the effect was small. It only explained up to 5 % of phenotypic variation and only in two of the four trials. The three QTL together explained up to 70 % of phenotypic variation for HPR. The *Q.DB.ui*-*7DS* is a major QTL consistently detected in all trials and explained up to 56 % of HPR variation. The *wPt*-*2565*-derived *STS* markers were present in 16 of 24 resistant lines derived from the three resistant sources PI 178383, CI 14106, and PI 476212. These markers will be useful in transferring and verifying the linked HPR alleles to assist in developing improved cultivars.

The three QTL are all new compared to the published HPR QTL for common bunt. Singh et al. (2016) reported a minor QTL on 7D for common bunt HPR derived from the resistance source, ‘Carberry’. Phenotypically, the Carberry QTL is substantially different than *Q.DB.ui*-*7DS* as the Carberry 7D HPR was negligible. The Carberry QTL, flanked by, *X664136* and *Xwmc273*, explained only 6 % of phenotypic variation. *X664136* and Xwmc273 span more than 26 cM and are unlinked to *Q.DB.ui*-*7DS* in the published DArT map (Wenzl et al. 2008). The *Q.DB.ui*-*7DS* is a novel QTL for dwarf bunt HPR in IDO444.

The pathogens of common and dwarf bunt are closely related, and resistance to both diseases in wheat is controlled by the same genes (Goates 2012). However, none of the IDO444-derived dwarf bunt HPR QTL align with published common bunt HPR QTL. IDO444 is a sib line of Blizzard; however, a Blizzard-derived QTL for common bunt was mapped on 1BS (Wang et al. 2009), while the IDO444—derived QTL were mapped on 1AS, 2B, and 7DS. Possibly the two sib lines, IDO444 and Blizzard, may carry different resistance genes to dwarf bunt (Goates, personal communication). However, given the sparse marker coverage on 7DS, it is possible that none of the 78 polymorphic SSR markers used in the Blizzard study were linked to the 7DS QTL detected in the present study. The two studies also used different inoculum sources: the Blizzard study used the common bunt races T-19 or T-19 and L-16, while this study used the composite dwarf bunt races. Additional common bunt HPR QTL have been reported from other resistance sources, including: 7A (Fofana et al. 2008; Dumalasová et al. 2012), 7B (Dumalasová et al. 2012; Knox et al. 2013), 5B (Dumalasová et al. 2012; Singh et al. 2016), 4D (Singh et al. 2016), and 6D (Menzies, et al. 2006; Singh et al. 2016), while none of these HPR QTL were detected in the present study. Singh et al. (2016) found that the common bunt HPR QTL co-localized with other beneficial traits including height and rust resistance. The three IDO444-derived QTL have no pleiotropic effect on other traits, such as coleoptile and root length (Li et al. 2011), stripe rust (Chen et al. 2012), grain yield, heading data, or height (Zhang et al. 2014) previously mapped in this population.

*Q.DB.ui*-*7DS* was identified in the Rio Blanco x IDO444 RIL population by the IDO444 haplotype for the DArT marker *wPt*-*2565* and the derived STS markers. However, that haplotype is also present in a number of additional lines with known putative resistance genes and also present in the differential lines for *Bt5*, *Bt8*, *Bt9*, *Bt10*, *Bt12*, and *Bt13* (Table 5). It is possible that the QTL contains one or more of these known HPR genes other than *Bt10*, as *Bt10* is already mapped to chromosome 6D (Menzies et al. 2006). The IDO444 haplotype is present in Promontory, while absent in Manning, Deloris, and Utah-100, and these cultivars all have the same putative HPR gene combination including *Bt*-*3*, *Bt*-*9*, and *Bt*-*10* (Table 5). Possibly, the marker and the QTL are no longer linked in coupling phase in these latter three cultivars. It is difficult to estimate the distance from the *wPt*-*2565* and derived STS markers to *Q.DB.ui*-*7DS* due to very low marker density but it is likely that this distance allows for recombination that breaks the haplotype–QTL linkage. This is an inherent limitation of marker-assisted selection when markers are not very tightly linked to the gene(s) of interest. Without knowledge of the pedigree and which parent is donating the appropriate haplotype linked to the QTL of interest it is not possible to reliably identify if HPR genes are present. However, the markers can be quite useful in a breeding program for bunt resistance where the parent pedigrees and flow of HPR alleles are known. Additional mapping studies will be necessary to identify additional QTL for dwarf and common bunt resistance, to resolve the resistances present in the differentials and in the most highly resistant cultivars, and to understand both bunt diseases and the corresponding resistances at a molecular level.

### Author contribution statement

ES conceived the study, generated the population, and contributed to marker identification and phenotyping; JC contributed to phenotyping, mapping, marker identification, and data analyses; MJG contributed to phenotyping, marker identification and data analyses; JZ contributed to mapping and data analyses; DH and BG contributed to phenotyping and data analyses. All authors contributed to, and approved the final manuscript.
